# Development of a Novel Real-Time Computer-Assisted Colonoscopy Diagnostic Tool for Colorectal Neoplasia and Cancer to Enable Personalized Patient Management: Protocol for a Multicenter Proof-of-Concept Study

**DOI:** 10.2196/77277

**Published:** 2026-10-21

**Authors:** Olaolu Olabintan, Angelo Brunori, Natalie Halvorsen, Nils Andersen, Verónica G Zubiaurre, Mehrnaz Mirzaei, Ina Dam Pedersen, Alessandro D’Aprano, Johnathan Sun, Ahmed Ghazwan Waraich, Omer Ahmad, Nastazja Pilonis, Michal Kaminski, Francisco Lamosa, Rune Erichsen, Johannes Blom, Thomas Rosch, Maria Pellise, Ignasi Puig, Cesare Hassan, Kensaku Mori, Yuichi Mori, Massashi Misawa, Shraddha Gulati

**Affiliations:** 1 King's Institute of Therapeutic Endoscopy, King's College Hospital, Denmark Hill, London, SE5 9RS, United Kingdom, 44 2032999000; 2 Faculty of Life Science and Medicine, King's College London, London, United Kingdom; 3 Fundació de Recerca Clínic Barcelona-Institut d’Investigacions Biomèdiques August Pi i Sunyer, Barcelona, Spain; 4 Department of Transplantation Medicine, Gastroenterology Section, Oslo University Hospital, Oslo, Norway; 5 Clinical Effectiveness Research Group, Institute of Health and Society, University of Oslo, Oslo, Norway; 6 Klinik und Poliklinik für interdisziplinäre Endoskopie, University Medical Center Hamburg-Eppendorf, Hamburg, Germany; 7 Digestive Disease Department, Althaia, Manresa, Spain; 8 Department of Internal Medicine, Stockholm South General Hospital, Stockholm, Sweden; 9 Department of Clinical Science and Education, Karolinska Institutet, Stockholm, Sweden; 10 Bedøvelse, Operation og Intensiv, Regional Hospital Horsens, Midtjylland, Denmark; 11 Department of Biomedical Sciences, Humanitas University, Milan, Italy; 12 Gastroenterology Department, University College Hospital, London, United Kingdom; 13 Department of Oncological Gastroenterology, National Research Institute of Onclolgy, Warsaw, Poland; 14 Maplesoft Europe, Duxford, United Kingdom; 15 Department of Surgery, Stockholm South General Hospital, Stockholm, Sweden; 16 Facultat de Medicina, Universitat de Vic - Universitat Central de Catalunya, Vic, Spain; 17 Institut de Recerca i Innovació Educativa, Vic, Spain; 18 IRCCS Humanitas Research Hospital, Milan, Italy; 19 Graduate School of Informatics, Nagoya University, Nagoya, Japan; 20 Digestive Disease Centre, Showa University Northern Yokohama Hospital, Yokohama, Japan

**Keywords:** artificial intelligence, AI, endoscopy, computer-assisted diagnosis, CADx, colorectal polyps

## Abstract

**Background:**

Accurate real-time classification of colorectal polyps during colonoscopy is essential for risk stratification, therapeutic decision-making, and surveillance planning, with important implications for colorectal cancer prevention, health care costs, and patient burden. Despite advances in optical diagnosis techniques, including virtual chromoendoscopy and magnification, widespread adoption remains limited because of high operator dependency and variable diagnostic accuracy. Existing computer-assisted diagnosis (CADx) systems predominantly provide binary classification of colorectal lesions, thereby constraining their clinical utility for nuanced endoscopic decision-making.

**Objective:**

This study aims to develop and validate a real-time, multiclass AI-based CADx system capable of classifying colorectal lesions into 5 categories: nonneoplastic lesions, low-grade adenomas, high-grade adenomas, sessile serrated lesions (SSL), and invasive colorectal cancer.

**Methods:**

This multicenter international study consists of 2 phases: model development and proof-of-concept validation. During the model development phase, a large, diverse dataset of >17,000 deidentified endoscopic images and video frames will be prospectively collected from 8 European centers and 1 Japanese center, capturing a broad spectrum of lesion types and patient demographics. A semisupervised learning framework using contrastive representation learning (SimCLR [simple framework for contrastive learning of visual representations]) will be used to leverage both labeled and unlabeled data, enhancing feature extraction and classification performance. Expert consensus annotation will be used for labeled data. Lesion localization will be supported by an integrated computer-assisted detection (CADe) module, while model interpretability will be facilitated through gradient-weighted class activation mapping (Grad-CAM). Proof-of-concept validation will be conducted using 250 previously unseen lesion images that will be independently assessed by expert endoscopists. The primary outcome is multiclass diagnostic accuracy, with a predefined target accuracy threshold of 85%, alongside sensitivity and specificity for invasive cancer detection. Secondary outcomes include interobserver agreement, performance across demographic and lesion subgroups, and a qualitative analysis of misclassification patterns. All data will be securely managed within a REDCap database, with strict deidentification protocols and plans to generate an open-access research resource.

**Results:**

Ethics approval has been obtained at all participating centres. Data transfer agreements have been executed by the majority of collaborating sites, with de-identified polyp images and videos currently being uploaded to a secure central database.

**Conclusions:**

This study aims to deliver a clinically applicable, real-time multiclass CADx system that extends beyond current binary AI models and supports more granular, personalized endoscopic decision-making. By integrating semisupervised learning, diverse international data, and explainable AI techniques, this approach has the potential to substantially advance the methodological foundations and clinical utility of AI-assisted optical diagnosis in routine colonoscopy, contributing to improved colorectal cancer prevention and patient care.

## Introduction

Accurate classification and characterization of polyps identified during colonoscopy are imperative for assessing colorectal cancer risk. Reliable identification of the malignant potential of individual polyps helps determine the most appropriate treatment strategy, while patient-level assessment of future colorectal cancer risk informs the selection of appropriate surveillance intervals (Figure 1). Accurate risk stratification at both the polyp and patient levels can substantially reduce the health care economic burden by decreasing the need for pathological assessments, increasing the use of organ-preserving endoscopic resection over surgical resection, and reducing the number of surveillance procedures and follow-up visits required. Consequently, this approach may also lessen the burden on patients [1].

**Figure 1.**
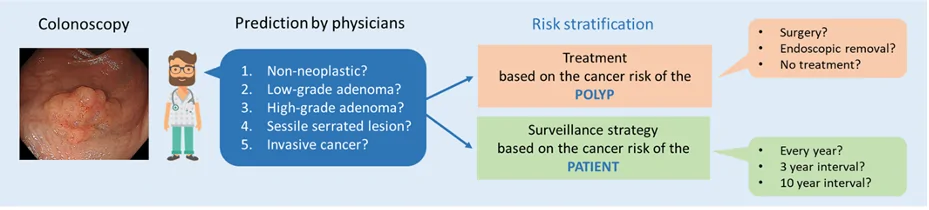
Overview of risk stratification–based clinical decision-making.

In the last decade, endoscopy societies have recommended visual assessment of polyp morphology to predict polyp pathology for risk stratification [2,3]. This visualization, or so-called optical diagnosis, often requires advanced imaging techniques such as virtual chromoendoscopy, magnification, and high-frequency miniprobe ultrasound, which require dedicated skills and expertise with an associated learning curve and increased procedure time [4]. For these reasons, despite being introduced into clinical practice, the uptake of such optical diagnosis techniques has been limited [5,6]. In addition to their low adoption rate, optical diagnosis accuracy has been reported as suboptimal in several real-world prospective studies [3]. For example, approximately 25% of surgically removed colorectal lesions are found to be benign adenomas, largely because of incorrect prediction during optical diagnosis [7].

To overcome this barrier and improve optical diagnosis, computer-assisted diagnosis (CADx) using AI technologies has recently attracted attention [8,9]. There are several commercially available CADx tools used in endoscopy practice, providing acceptable accuracy for optical diagnosis [8,10]. However, currently available CADx platforms differentiate only between neoplastic and nonneoplastic lesions, which we believe limits personalized patient risk stratification [11,12]. In particular, the inability to identify cancer and sessile serrated lesions (SSLs) negatively impacts the flexible use of CADx in colonoscopy practice. Colorectal cancer requires a different treatment approach from adenomas, while SSLs should be handled as neoplasia even though they often look quite similar to hyperplastic polyps, which require neither treatment nor surveillance. Therefore, the development of a multiclass algorithm is of greater interest and relevance than the currently available binary classification system. As part of the European Union–supported OperA project (Horizon Europe 101057099), we plan to develop a CADx tool to differentiate among multiple pathological categories, including nonneoplastic lesions, low- and high-grade adenomas, SSLs, and colorectal cancers. These categories align with varying treatment options and current surveillance guidelines [13,14].

## Methods

### Study Design

This is a multicenter, international study. The study includes 2 phases, namely, the development and test phases. In the development phase, we will collect deidentified static images and videos for the initial development of the AI algorithm. These data will be used to develop the CADx tool. In the test phase, we will validate the performance of the CADx tool using an image-based benchmark test.

### Data Collection: Global-Level Data Accumulation

Data will be collected prospectively (2023-2025) and retrospectively (2000-2024). Even though there is a chronological difference between these approaches, both follow the same inclusion and exclusion criteria for patient inclusion (Textbox 1). Data collection will be conducted as part of patients’ usual care and will not affect their treatment pathway. Data collected retrospectively will be used exclusively for the development of the CADx tool, while the data collected prospectively will be used for either the development or validation of the CADx tool.

The development of a 5-class CADx (classifying nonneoplastic lesions, low- and high-grade adenomas, SSLs, and cancers) tool requires a larger number of images than the current binary CADx classification system. As a basis for the dataset, fully anonymized data from existing image libraries established in previous studies by the investigators in Japan will be used [15,16]. The library contains >100,000 image frames collected in Japan, of which at least 10,000 images will be used for this study.

To add further diversity and volume, we will include data from another 10 European hospitals in Norway, the United Kingdom, Poland, Denmark, Sweden, Germany, and Spain. The inclusion of at least 7000 lesions from these countries is planned (Table 1), with paired pathological diagnoses obtained through resection or biopsy.

Textbox 1. **Inclusion and exclusion criteria.**

**Inclusion criteria**

- Age ≥18 years
- Undergoing symptomatic or screening colonoscopy

**Exclusion criteria**

- Inability to provide informed consent
- Colitis-associated dysplasia
- Polyps at a surgical anastomosis
- Pregnancy

**Table 1.** Participating countries, hospitals, and lesions included in the machine learning and validation study.

Countries	Hospitals (n=16), n (%)	Lesions (n=17,000), n (%)
Norway	2 (12.5)	1000 (6)
United Kingdom	2 (12.5)	1000 (6)
Poland	2 (12.5)	1000 (6)
Denmark	2 (12.5)	1000 (6)
Sweden	2 (12.5)	1000 (6)
Germany	2 (12.5)	1000 (6)
Spain	2 (12.5)	1000 (6)
Japan	2 (12.5)	10,000 (58)

In total, 17,000 lesions will be used for machine learning. Among these, we aim to include as many T1 cancers as possible (at least 2000 lesions) to optimize the AI’s discrimination ability for this important category. Combining data from Eastern and Western populations contributes not only to greater lesion variability but also to increased ethnic diversity, both of which are indispensable for a high-performance AI model.

In the retrospective data collection, we will collect only 2 types of information, namely, endoscopic images or videos and the corresponding pathology. On the other hand, in the prospective data collection, we will also record patient demographic data and detailed polyp-level data, including sex, age, colonoscopy indication; patient ethnicity; and polyp location, size, and morphology.

### Data Training Phase: Semisupervised Learning Algorithm

A semisupervised learning methodology will be used to deliver a reliable 5-class CADx model. This process includes the following steps.

#### Step 1: Creating Labeled and Unlabeled Datasets

The creation of labeled and unlabeled datasets will involve the following procedures:

- A review of all 17,000 lesions will be conducted by 3 experienced endoscopists (each with experience performing >3000 colonoscopies).
- An image will be tagged as labeled if the 3 experts agree on the preassigned pathological diagnosis of the lesion.
- If there is disagreement regarding the preassigned diagnosis, the pathological diagnosis will be removed from the image, and the image will be tagged as unlabeled data.

#### Step 2: Contrastive Learning

To construct the optimal algorithm architecture, a 2-step semisupervised training framework named SimCLR (simple framework for contrastive learning of visual representations) will be adopted (Figure 2) [17]. The first phase is the self-supervised pretraining phase, during which contrastive learning using unlabeled data is conducted. The second phase is the supervised (fine-tuning) phase, during which the pretrained model is fine-tuned using the labeled data.

**Figure 2.**
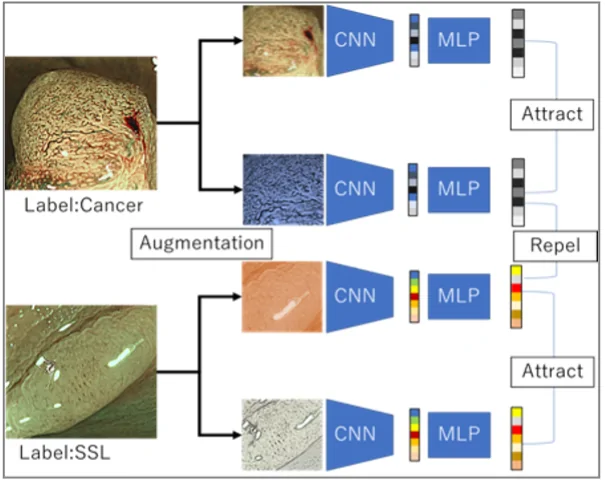
Schematic overview of the 2-step semisupervised learning framework. CNN: convolutional neural network; MLP: multilayer perceptron; SSL: sessile serrated lesion.

Contrastive learning leverages the principle that images sharing the same diagnosis will yield similar feature representations when subjected to augmentation [17]. Specifically, each image undergoes 2 distinct transformations, generating a pair of augmented views. These views are processed through a convolutional neural network (CNN) encoder and a multilayer perceptron (MLP) projection head to produce embeddings [18,19]. The training objective is to minimize the distance between embeddings of the same image (positive pairs) while maximizing the distance between embeddings of different images (negative pairs). This approach encourages the model to learn discriminative features that cluster similar instances and separate dissimilar instances in the embedding space [4,18].

During the pretraining phase with unlabeled data, both the CNN and MLP are optimized to minimize a contrastive loss function, such as the information noise-contrastive estimation (InfoNCE) loss, which quantifies the similarity between positive and negative pairs in the learned representation space. After the pretraining phase, the CNN encoder can be fine-tuned for downstream tasks by appending a fully connected classification layer, enabling effective performance even with limited labeled data [4,18].

### Integration of Computer-Assisted Detection Architecture and Interpretable AI

The semisupervised CADx model should be activated only when a lesion is detected. Therefore, a conventional computer-assisted detection (CADe) algorithm, developed as part of preliminary work (a YOLO version 3–based fully supervised learning model trained on 56,668 images), will be integrated into the final model [16].

To provide an interpretable output from the CNN, gradient-weighted class activation mapping (Grad-CAM) will be used (Figure 3). This method generates a heat map image in which the pixels that are important for the prediction are highlighted. Physicians can identify the mislocalization of the algorithm when the highlighted pixels are located outside the polyp. The predicted probability will also be shown for transparency. All machine learning activities will be conducted collaboratively by King’s College, Showa University, Nagoya University, and Universitat Autònoma de Barcelona, all of which have extensive experience in developing deep learning–based prediction models for colonoscopy [11,20].

**Figure 3.**
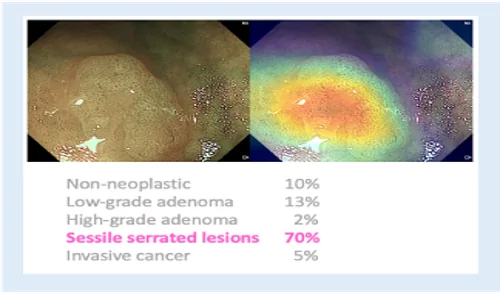
Gradient-weighted class activation mapping (Grad-CAM) with percentage probabilities for each diagnostic category.

### Proof-of-Concept Study and Sample Size Calculation

Proof-of-concept validation will be performed using 250 previously unseen lesions evenly distributed across the 5 diagnostic categories to enable controlled, class-balanced benchmarking of multiclass discrimination. The performance will be evaluated primarily using sensitivity and specificity for invasive cancer detection, while overall multiclass accuracy across the 5 diagnostic categories will be reported as a secondary feasibility metric.

### Data Management

No clinical data will be collected on a paper format; all data are entered directly into a password-protected database enabled through the REDCap (Vanderbilt University) web-based application hosted by King’s College London University. This database will only be accessible to the trained research staff. All data entered into the database are all deidentified. If required, patient information will be accessed through the password-protected electronic health record maintained as part of the participant’s routine medical care.

### Open-Access Database

Through this multicenter cohort study, we will generate a large, fully deidentified database of still images and videos. The intention is to promote further studies on AI and colonoscopy. Researchers outside this study will be able to use the open data source, which can result in diverse and innovative ideas to answer several research questions. In this database, we will include still images and videos from 1000 diverse colorectal lesions. No personally identifiable information will be included.

### Dissemination and Implementation

Findings from this study will be disseminated through peer-reviewed publications, international gastroenterology conferences, and engagement with professional societies. Where appropriate, results will be shared with regulatory bodies and the clinical guidelines development team to inform evidence-based adoption.

### Ethical Considerations

For the prospective data collection, eligible patients will be identified approximately 7 days before their procedure. The patient information leaflet and consent form will be sent to patients by post, email, or telephone message, according to their preference. A member of the research team will see the patient on the day of the procedure to speak to the patient and address any concerns, before obtaining the written informed consent. For the retrospective data collection, if a pre-existing consent is not available for stored images to be used for research purposes, a text will be sent to all patients informing them about the research, and an opportunity will be given to the patient to opt out within a 2-week period. Each hospital will obtain ethics approval from its research ethics committee before data collection starts. In the United Kingdom, ethics approval was granted by Health and Care Research Wales (24/LO/0176).

### Outcomes and Data Analysis

#### Overview

A proof-of-concept study will be performed when the prototype AI algorithm has been developed. The study involves benchmark testing using 250 randomly selected, previously unseen endoscopic images from 250 lesions with paired pathological diagnoses and diagnostic assessments by 10 expert endoscopists and 10 nonexpert endoscopists. The 5 categories will be equally represented, with 50 lesions selected from each category. The threshold for a successful algorithm has been set at 85%, as the maximum reported accuracy achieved by physicians for multiclass classification of colorectal polyps is approximately 80% [21].

#### Study Outcomes

##### Primary Outcomes

The primary outcomes include sensitivity and specificity for predicting cancer pathology.

##### Secondary Outcomes

The secondary outcomes are as follows:

- Sensitivity and specificity for predicting nonneoplastic lesions, low- and high-grade adenomas, and SSLs
- Interobserver agreement among the endoscopists regarding the predicted histology of polyps during the annotation process
- Subgroup analysis of polyp characteristics focused on different genders and ethnicities
- Assessment of whether patient gender, ethnicity, and age can be predicted using the developed CADx model. This will be evaluated in a subgroup analysis of an AI model trained on the prospective data cohort. Performance will be measured using accuracy and the area under the receiver operating characteristic curve for each gender, ethnicity, and age group.
- A qualitative analysis of incorrect CADx diagnoses conducted by a multidisciplinary panel to evaluate their potential impact
- Evaluation of the effects of AI-augmented endoscopy on endoscopists’ optical diagnosis (human-AI interaction, exploratory and descriptive, intended to inform the design of future prospective interventional studies)

## Results

Ethics approval has been obtained at all participating centres, and data transfer agreements have been completed by the majority of collaborating institutions. Participating sites are actively uploading de-identified polyp images and videos to a secure central database. Data acquisition remains ongoing, with quality assurance processes in place to verify image quality, annotation accuracy, histopathology correlation, and completeness of accompanying clinical metadata before inclusion in the development and validation datasets. Recruitment and data acquisition are progressing as planned, with completion of the proof-of-concept study anticipated by December 2026.

## Discussion

### Anticipated Findings

This study is designed to develop and validate a real-time, multiclass CADx system capable of characterizing colorectal lesions into clinically relevant categories. The anticipated primary outcome is the demonstration that a 5-class classification framework can achieve robust diagnostic performance across a diverse, multinational dataset while addressing key limitations of existing binary CADx models. By incorporating low- and high-grade adenomas, hyperplastic polyps, SSLs, and early invasive cancer into a unified framework, the proposed system is expected to provide more granular and clinically actionable information at the point of care.

In addition, the integration of CADe and explainability tools is anticipated to support reliable lesion localization and enhance clinicians’ understanding of model outputs. Together, these components aim to facilitate safe and clinically appropriate application of AI-assisted diagnosis in routine colonoscopy.

Most commercially available and investigational CADx systems currently focus on binary classification of neoplastic vs nonneoplastic lesions [22], primarily to support the “resect and discard” or “leave in situ” strategies for diminutive polyps [23]. Although these approaches have demonstrated high accuracy in controlled settings [22], they provide limited guidance for more complex lesions, particularly SSLs and early cancers.

Recent studies exploring multiclass classification have shown promise but have often been constrained by small datasets, limited lesion diversity, or single-region training cohorts [24]. In contrast, our study builds on the emerging literature by incorporating a large, ethnically diverse dataset from multiple geographical regions and by including substantial representation of different lesion types. The use of semisupervised contrastive learning also extends prior work by reducing dependence on fully annotated datasets, addressing a recognized barrier to scalable AI development in endoscopy.

Furthermore, although previous CADx studies have primarily focused on diagnostic accuracy [8,10], fewer have systematically addressed interpretability and clinical integration [22]. The inclusion of Grad-CAM represents an important step toward aligning algorithmic output with clinician reasoning and guideline expectations.

A major strength of this approach is the use of a large, ethnically diverse, multinational dataset sourced from both Eastern and Western populations. This design directly addresses concerns regarding limited generalizability and dataset bias that have constrained prior CADx systems. The inclusion of a substantial number of SSLs and early colorectal cancers (T1 cancers) further strengthens the model’s relevance, given the recognized diagnostic challenges associated with these entities. Methodologically, the use of semisupervised contrastive learning represents an important advance, allowing effective use of unlabeled data and reducing reliance on exhaustive expert annotation—an acknowledged bottleneck in medical AI development.

Integration of a CADe module ensures that diagnostic predictions are contextually triggered by lesion localization, while the incorporation of Grad-CAM enhances model interpretability. This focus on explainability is particularly important for clinician trust, governance, and future regulatory acceptance of AI-enabled diagnostic systems.

Several limitations merit consideration. Proof-of-concept validation is image-based and does not fully replicate real-time procedural conditions, including motion artifacts, workflow pressures, and human-AI interaction.

Second, although expert consensus annotation is used, interobserver variability remains an inherent challenge in optical diagnosis and may influence model training. Finally, as this is a development and validation study, downstream effects on clinical decision-making, surveillance intervals, and patient outcomes cannot be assessed within the scope of the current protocol.

These limitations address the need for cautious interpretation of the findings and highlight that clinical effectiveness must be established through subsequent prospective studies.

Future work will focus on prospective real-time validation in routine clinical settings, with emphasis on workflow integration, user interaction, and safety monitoring. Additional research will be required to assess long-term outcomes, including changes in surveillance adherence, resource use, and cost-effectiveness.

### Conclusions

This study is the first to develop a 5-class AI algorithm for real-time histological prediction of colorectal polyps, advancing beyond the binary classification used in current CADx systems. By enabling more detailed risk stratification, the tool aims to support accurate treatment decisions, including onward referral to specialized centers, and guide appropriate surveillance intervals. Trained on a large, ethnically diverse dataset from 8 countries, the algorithm is designed to be generalizable and applicable across varied clinical settings. This proof-of-concept study has the potential to enhance the utility of CADx in routine endoscopic practice by providing real-time, personalized pathology predictions to support clinical decision-making.

## Abbreviations

CADe: computer-assisted detection
CADx: computer-assisted diagnosis
CNN: convolutional neural network
Grad-CAM: gradient-weighted class activation mapping
InfoNCE: information noise-contrastive estimation
MLP: multilayer perceptron
SimCLR: simple framework for contrastive learning of visual representations
SSL: sessile serrated lesion
